# RIG-1-Like Receptor Activation Synergizes With Intratumoral Alpha Radiation to Induce Pancreatic Tumor Rejection, Triple-Negative Breast Metastases Clearance, and Antitumor Immune Memory in Mice

**DOI:** 10.3389/fonc.2020.00990

**Published:** 2020-07-17

**Authors:** Vered Domankevich, Margalit Efrati, Michael Schmidt, Eran Glikson, Fairuz Mansour, Amit Shai, Adi Cohen, Yael Zilberstein, Elad Flaisher, Razvan Galalae, Itzhak Kelson, Yona Keisari

**Affiliations:** ^1^Department of Clinical Microbiology and Immunology, Sackler Faculty of Medicine, Tel Aviv University, Tel Aviv-Yafo, Israel; ^2^Alpha Tau Medical, Tel Aviv-Yafo, Israel; ^3^Sackler Faculty of Exact Sciences, School of Physics and Astronomy, Tel Aviv University, Tel Aviv-Yafo, Israel; ^4^Department of Otolaryngology, Head and Neck Surgery, Sheba Medical Center, Tel HaShomer, Israel; ^5^Sackler Cellular and Molecular Imaging Center, Sackler Faculty of Medicine, Tel Aviv University, Tel Aviv-Yafo, Israel; ^6^MedAustron, Wiener Neustadt, Austria; ^7^Medical Faculty, Christian-Albrechts University, Kiel, Germany

**Keywords:** PolyIC, polyethylenimine, decitabine, radiotherapy, immunotherapy, alpha radiation, triple-negative, breast carcinoma

## Abstract

Diffusing alpha-emitting radiation therapy (DaRT) employs intratumoral Ra-224-coated seeds that efficiently destroy solid tumors by slowly releasing alpha-emitting atoms inside the tumor. In immunogenic tumor models, DaRT was shown to activate systemic antitumor immunity. Agonists of the membrane-bound toll-like receptors (TLRs) enhanced these effects and led to tumor rejection. Here, we examined the combination of DaRT with agents that activate a different type of pattern recognition receptors, the cytoplasmatic RIG1-like receptors (RLRs). In response to cytoplasmatic viral dsRNA, RLRs activate an antiviral immune response that includes the elevation of antigen presentation. Thus, it was postulated that in low-immunogenic tumor models, RLR activation in tumor cells prior to the induction of their death by DaRT will be superior compared to TLR activation. Intratumoral cytoplasmatic delivery of the dsRNA mimic polyIC by polyethylenimine (PEI), was used to activate RLR, while polyIC without PEI was used to activate TLR. PolyIC(PEI) prior to DaRT synergistically retarded 4T1 triple-negative breast tumors and metastasis development more efficiently than polyIC and rejected panc02 pancreatic tumors in some of the treated mice. Splenocytes from treated mice, adoptively transferred to naive mice in combination with 4T1 tumor cells, delayed tumor development compared to naïve splenocytes. Low-dose cyclophosphamide, known to reduce T regulatory cell number, enhanced the effect of DaRT and polyIC(PEI) and led to high long-term survival rates under neoadjuvant settings, which confirmed metastasis clearance. The epigenetic drug decitabine, known to activate RLR in low doses, was given intraperitoneally prior to DaRT and caused tumor growth retardation, similar to local polyIC(PEI). The systemic and/or local administration of RLR activators was also tested in the squamous cell carcinoma (SCC) tumor model SQ2, in which a delay in tumor re-challenge development was demonstrated. We conclude that RIG-I-like activation prior to intratumoral alpha radiation may serve as a potent combination technique to reduce both tumor growth and the spread of distant metastases in low-immunogenic and metastatic tumor models.

## Introduction

The destruction of the live tumor inside the host (namely, tumor ablation) releases tumor antigens to the tumor microenvironment and stimulates the activation of systemic and specific antitumor immune responses. Accordingly, tumor ablation can be considered as a form of “*in situ* vaccination” against tumor cells (1–4). Consequently, tumor ablation treatments may achieve two important goals simultaneously: [1] the destruction of the primary tumor and [2] the activation of antitumor immune responses against residual and distant tumor cells. This contrasts with surgical tumor resection, which achieves the first goal yet may suppress the second (5–7).

A unique radiotherapeutic tumor ablation technique utilizing the diffusion of alpha emitting atoms inside the tumor (diffusing alpha-emitters radiation therapy, referred to as DaRT henceforth) was shown to efficiently destroy a wide range of solid tumors, while sparing the adjacent tissues. This technique utilizes Radium (^224^Ra)-loaded stainless-steel wires or tubes (DaRT seeds) that release daughter atoms inside the tumor to a range of several millimeters (8–14). DaRT was shown to activate systemic immune memory when used as a monotherapy (15, 16). When combined with immunoadjuvants and inhibitors of immune suppressive cells, DaRT led to long-term rejection of immunogenic tumors in mice, whereas the same immunomodulatory treatment with a non-radioactive seed mostly led to tumor recurrence (17, 18). Long-term rejection of tumors was correlated with a specific immune memory against tumor antigens (18), suggesting that cell death by alpha radiation activates tumor antigen recognition at the ablation site. This agrees with reports showing that the cell response to radiation includes the elevation of damage-associated molecular patterns (DAMPs) (19), MHC class I expression (20), and interferon responses (21) that may contribute to antigen presentation, cross presentation, APC activation, and recruitment of effector T lymphocytes (22).

Another type of *in situ* vaccination employs the activation of cytoplasmatic viral sensors such as RIG-I-like receptors (RLRs). RLRs (e.g., RIG-I and MDA5) sense cytoplasmatic viral dsRNA as part of a conserved defense mechanism of the innate immune system (22–24). Upon activation, these sensors promote antigen presentation, a type-1 interferon response, pyroptosis, DAMPs secretion, and immunogenic cell death (25). Recently, RLR activation was found to boost the efficiency of anticancer vaccines and to be critical for responsiveness to immune checkpoint blockade (26, 27).

One way to activate RLRs is to deliver a dsRNA viral mimic, such as polyIC, directly into the cytoplasm of the cell, while bypassing endosomal recognition via toll-like receptors, such as TLR3 (28). This is done using a delivery agent, such as the cationic polymer polyethylenimine (PEI), which masks the viral dsRNA until it reaches the cytoplasm, where it is released and recognized (29, 30). PolyIC cytoplasmic delivery was previously tested as a targeted therapy (26, 31), a systemic therapy (32, 33), and a local therapy (34). However, the effects of this treatment on distant metastases or in combination with radiotherapy have not been well-investigated. Interestingly, hallmarks of immunogenic cell death were observed in tumor cells following treatment with polyIC complexed with PEI, including the elevation of MHC class I expression. However, identical concentrations of polyIC (a TLR3 agonist) without PEI failed to elevate MHC class I expression (3), suggesting that the RLR pathway is superior to the TLR pathway with regard to antigen presentation on tumor cells following activation.

Another way to stimulate RLRs is by using DNA methyltransferase (DNMT) inhibitors such as decitabine (35). DNMT inhibitors can stimulate endogenous retroviruses that are sensed by RLRs (36–38) or inhibit the methylation of RLR genes promoters (35). RLR activation by both cytoplasmatic delivery of dsRNA (34) and DNMT inhibitors (39–41) was shown to upregulate MHC class I and to potentiate interferon and cytotoxic T lymphocyte responses.

Both alpha radiation-based ablation (16) and RLR activation (34) were shown to induce local tumor cell killing and a systemic antitumor response. It was shown that radiation-mediated antitumor immunity requires a cytosolic DNA-sensing pathway, such as the stimulator of interferon genes (STING) pathway (42). The fact that DNA-sensing and RNA-sensing function via different pathways may increase the potential to achieve a synergy between DaRT and RLR activation.

The current study investigated a novel approach to combine alpha radiation-based ablation and RLR activation in low-immunogenic and metastatic tumor models, such as the triple negative breast cancer (TNBC) mouse model 4T1, the pancreatic carcinoma tumor model Panc02, and the squamous cell carcinoma (SCC) tumor model, SQ2. Aggressive tumors such as TNBC and pancreatic cancer demonstrate low immunogenicity, which correlates with low responsiveness to immunotherapy and is mainly determined by tumor antigenicity and antigen presentation efficiency (43). MHC class I molecules on the surface of tumor cells were identified as critical for the enhancement of immunotherapy effectiveness (44). In support of this, it was recently demonstrated that antigens presented in the context of MHC class I, pulled down from tumor cell lysate, can serve as an artificial antigen presenting cell and induce potent and specific effector CD8+ T cell responses against tumor cells (45). In the current study, RLR activation was used long enough prior to the induction of cell death by alpha radiation to allow the potential enhancement of antigen presentation on tumor cells, which may be crucial for achieving antigen-specific antitumor immunity in low-immunogenic tumors. The effect of the treatment on tumor development and on metastatic load was investigated by probing lung metastases at a late timepoint. In addition, long-term survival after local treatment and tumor resection was used to confirm clearance of metastases. Immune memory was investigated by employing the Winn and challenge assays. Finally, the treatment was combined with systemic immunomodulation.

## Materials and Methods

### Animals

All animal experiments were carried out in accordance with the government and institution guidelines and regulations (Ethics approval IDs 01-18-030, 01-19-039, 01-19-081) and with the National Institutes of Health guide for the care and use of Laboratory animals (NIH Publications No. 8023, revised 1978). BALB/c and C57BL/6 female mice (~20 g, 10 weeks old) were obtained from Envigo (Jerusalem, Israel) and were kept in the animal facility of Tel Aviv University. All surgical and invasive procedures were performed under anesthesia using ketamine (100 mg/kg, Bremer Pharma, Germany) and xylazine hydrochloride (10 mg/kg, Eurovet Animal Health B.V., Bladel, Netherlands) solution in PBS. Intraperitoneal inoculation was given 10 min before starting the treatment.

### Tumor Cell Lines

All cell lines were incubated in a humid incubator at a temperature of 37°C and 5% CO_2_. M-cherry-labeled 4T1 mammary adenocarcinoma tumor cells (kindly provided by Prof. Satchi-Fainaro, Faculty of Medicine, Tel Aviv University, Tel Aviv, Israel) were grown in RPMI-1640 containing L-glutamine, supplemented with 10% fetal calf serum, penicillin (100 U/ml), streptomycin (100 μg/ml), nystatin (12.5 U/ml), sodium pyruvate (1 mM), and HEPES buffer 1 M (Biological Industries, Kibbutz Beit Haemek, Israel). Panc02 murine pancreatic carcinoma (kindly provided by Dr. Hollingsworth, Eppley Institute, Nebraska University Medical Center, USA) were grown in Dulbecco's modified eagle medium (DMEM) supplemented with 10% fetal calf serum, penicillin (100 U/ml), streptomycin (100 μg/ml), nystatin (12.5 U/ml), sodium pyruvate (1 mM), and MEM Non-Essential Amino Acids (Biological Industries, Kibbutz Beit Haemek, Israel). SQ2 murine squamous cell carcinoma (kindly provided by Dr. Gad Lavie from the Sheba Medical Center, Tel HaShomer, Israel) were grown in Dulbecco's modified eagle medium (DMEM) supplemented with 10% fetal calf serum, penicillin (100 U/ml), streptomycin (100 μg/ml), and nystatin (12.5 U/ml) (Biological Industries, Kibbutz Beit Haemek, Israel).

### Tumor Cell Inoculation

4T1^MCherry^, SQ2, and panc02 tumor cells were inoculated in doses of 2.5 × 10^5^, 5 × 10^5^, and 6 × 10^5^, respectively. Mice were inoculated intracutaneously into the right (unless stated otherwise) low lateral side of the back in 0.05 mL Hanks' balanced salt solution (HBSS, Biological Industries, Kibbutz Beit Haemek, Israel).

### Drug Preparations

According to previous studies, high-molecular-weight (HMW) polyIC induced stronger immune activation than low-molecular-weight (LMW) polyIC (46) and was therefore chosen to be delivered into tumor cells in the current study. PolyIC HMW VacciGrade™ (InvivoGen, USA) was prepared in aliquots according to manufacturer instructions and kept at −20°C. At the day of treatment, polyIC was mixed with *in vivo*-jetPEI® (Polyplus, France) according to manufacturer instructions. Briefly, polyIC and PEI were diluted in 5% glucose solution and incubated at a ratio of N:P = 6 for 15 min at room temperature. PolyIC was intratumorally injected to the tumor 72 and 24 h prior to DaRT insertion. 5% glucose served as vehicle unless mentioned otherwise. Cyclophosphamide (Sigma C0768, Israel) was prepared at the indicated concentrations in saline solution. CP was administrated i.p. in the dose of 100 mg/kg 24 h prior to polyIC. Decitabine (Tocris, UK) was prepared in PBS. Decitabine was administrated i.p. in the dose of 1 mg/kg daily for 4 consecutive days prior to DaRT insertion.

### ^224^Ra-Loaded Seed Preparation and Insertion

Stainless steel (316 LVM) 0.7-mm-diameter tubes in the length of 6.5 mm (unless mentioned otherwise) were loaded with ^**224**^Ra atoms, following an electrostatic collection process similar to that described in (12). To prevent radium detachment from the surface, the seeds were coated, in this study, with a 250-nm (nanometer) polymeric layer (Nusil, med2-4213 model). The ^220^Rn desorption probability (the probability that a ^220^Rn atom is emitted from the seed following a decay of ^224^Ra) was 45% (unless mentioned otherwise). The ^224^Ra activity in kBq is indicated for each experiment in the Results section. Seeds, either loaded with ^**224**^Ra or inert, were placed near the tip of a 19-gauge needle, which is attached to an insertion applicator. The radioactive and inert seeds were inserted into the tumor under anesthesia.

### *In vivo* Tumor Measurements

Local tumor growth was determined by measuring 3 mutually orthogonal tumor dimensions 2–3 times per week, according to the following formula: Tumor volume = π/6 × Diameter 1 × Diameter 2 × Height. Daily survival monitoring was performed and recorded.

### Tumor and Metastasis Imaging and Analysis

CRI Maestro™ (Cambridge Research and Instrumentation, USA) was used to measure M-Cherry signal. Multispectral image cubes were acquired through a 550–800-nm spectral range in 10-nm steps using an excitation (595 nm longpass) and emission (645 nm longpass) filter set, under exposure time of 2,000 ms. Autofluorescence signals were eliminated by spectral analysis and linear unmixing algorithm of the CRI-Maestro software. Computed tomography (CT) scan was performed using the TomoScope Synergy microCT scanner (CT imaging, Erlangen, Germany) under anesthesia. Data was acquired using 360° individual projection collected every 1° to complete one rotation around the animal, with X-ray tube voltage of 40 kV. Cross-sectional images (DICOM format) were generated using TomoScope image reconstruction software (CT imaging, Germany) and were analyzed using “RadiAnt” software.

### Histology

For histological H&E staining, lungs were washed in PBS and fixed in a 4% formaldehyde solution (Bio-Lab, Jerusalem, Israel) for at least 24 h. The preserved specimens were processed in ethanol and xylene and then embedded in paraffin. Six-μm sections were then stained with hematoxylin (Sigma, Rehovot, Israel) and eosin (Surgipath, Richmond, VA, USA).

### Winn Assay

Spleens were harvested, immersed in PBS, ground with the flat end of a syringe, and passed through a cell strainer. Cells were washed in RPMI/HBSS and centrifuged at 394 × g for 7 min. The supernatant was removed, and cells were resuspended and pooled. Red blood cells were lysed, and cells were washed in HBSS. Cells were then mixed with tumor cells in the indicated ratio and immediately injected in a volume of 0.15 ml.

### Statistical Analysis

The difference between the mean values of two groups was determined by two-sided Student's T-test on the last day of the experiment, unless mentioned otherwise. The difference in the proportion of an event between two groups was determined by χ^2^ test. Differences in the survival period between two groups were determined by log-rank test. *p* < 0.05 was considered as significant difference between groups.

## Results

### Intratumoral polyIC^PEI^ and DaRT Synergistically Inhibit the Development of 4T1 Solid Tumors and Metastases

A previous study done in the immunogenic tumor model CT26 has shown that combining DaRT with TLR agonists led to long-term tumor rejection, which was not observed when DaRT was used alone (18). Here, it was investigated whether in the low-immunogenic tumor model 4T1 using RLR activation in combination with DaRT is superior to TLR activation, and whether this combination is synergistic in terms of long-term local and systemic retardation of tumor development. To answer these questions, intratumoral administration of the dsRNA viral mimic, polyIC, was used in two forms, as follows. Either complexed with the delivery polymer PEI (polyIC^PEI^) to enable the cytoplasmatic delivery of polyIC and the activation of the RIG-1 receptor MDA5 (47) or “Naked” polyIC (polyIC^naked^), which agonizes the toll-like receptor TLR3 (48).

**Figure 1:**
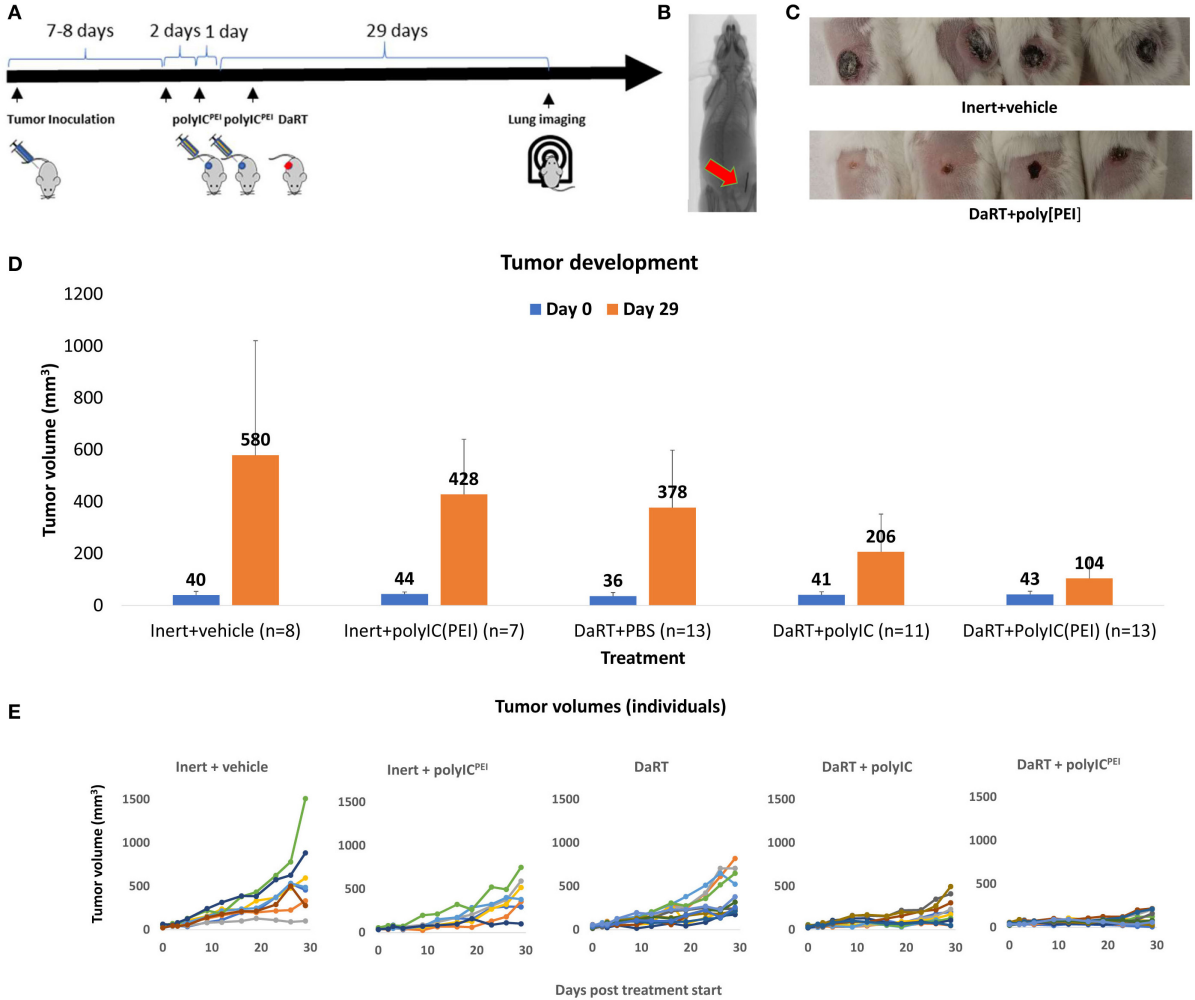
Primary tumor growth following treatment with DaRT+polyIC^PEI^. 4T1-bearing mice (40 mm^3^) were treated intratumorally with polyIC^PEI^ (20 μg/40 μl) or polyIC^naked^ (20 μg/40 μl) or vehicle, 72 and 24 h prior to the insertion of a single DaRT seed (length = 8 mm, activity = 70 kBq) or an inert seed (for 29 days). **(A)** Schematic representation of the treatment protocol. **(B)** CT image of a DaRT seed inside the tumor. **(C)** Representative primary tumors 29 days after tumor cell inoculation. **(D)** Mean tumor volumes ± SEM on the day of treatment start (0) and on the day of experiment termination (29). *p*_t−test_ < 0.005 for DaRT+ polyIC^PEI^ vs. control; *p*_t−test_ < 0.0005 for DaRT+ polyIC^PEI^ vs. DaRT alone; *p*_t−test_ < 0.0005 for DaRT+ polyIC^PEI^ vs. polyIC^PEI^ alone; *p*_t−test_ < 0.05 for DaRT+ polyIC^PEI^ vs. DaRT+polyIC^naked^. **(E)** Individual tumor growth curves for each treatment, up to 29 days from treatment start. Each line represents an individual mouse. The results are based on cumulative data from two different experiments.

Mice bearing 4T1 tumors were treated by an intratumoral injection of 20 μg/40 μl polyIC^PEI^, polyIC^naked^, or PBS followed by the insertion of a single DaRT seed (length = 8 mm, activity =70 kBq) or a non-radioactive (inert) seed. Twenty-nine days after treatment started, lungs were scanned by computed tomography (CT). The experiment was terminated 37–38 days following tumor cell inoculation, and lungs were imaged for M-Cherry fluorescent signal (Figures 1A,B).

The results indicated that DaRT combined with polyIC^PEI^ significantly retarded tumor growth (*p*_t−test_ < 0.05) compared to all other groups (Figures 1C–E). The percent reduction in tumor volume compared to inert+vehicle control was calculated for each treatment (according to the following formula: [(mean tumor volume at day 29 in the treatment group)/(mean tumor volume at day 29 of control group)−1] × 100). The cytoplasmatic delivery of polyIC treatment on its own reduced tumor size by 26% compared to inert+vehicle (control). Alpha radiation treatment on its own reduced tumor size by up to about 34% compared to control. The combination of alpha radiotherapy and cytoplasmatic delivery of the viral mimic polyIC reduced the tumor size by 82%, demonstrating a synergistic effect between the treatments. Treatment with DaRT+polyIC^naked^ significantly retarded tumor growth compared to DaRT alone or inert+vehicle control (Figures 1C,E). However, the treatment was significantly less effective compared to DaRT combined with polyIC^PEI^.

**Figure 2:**
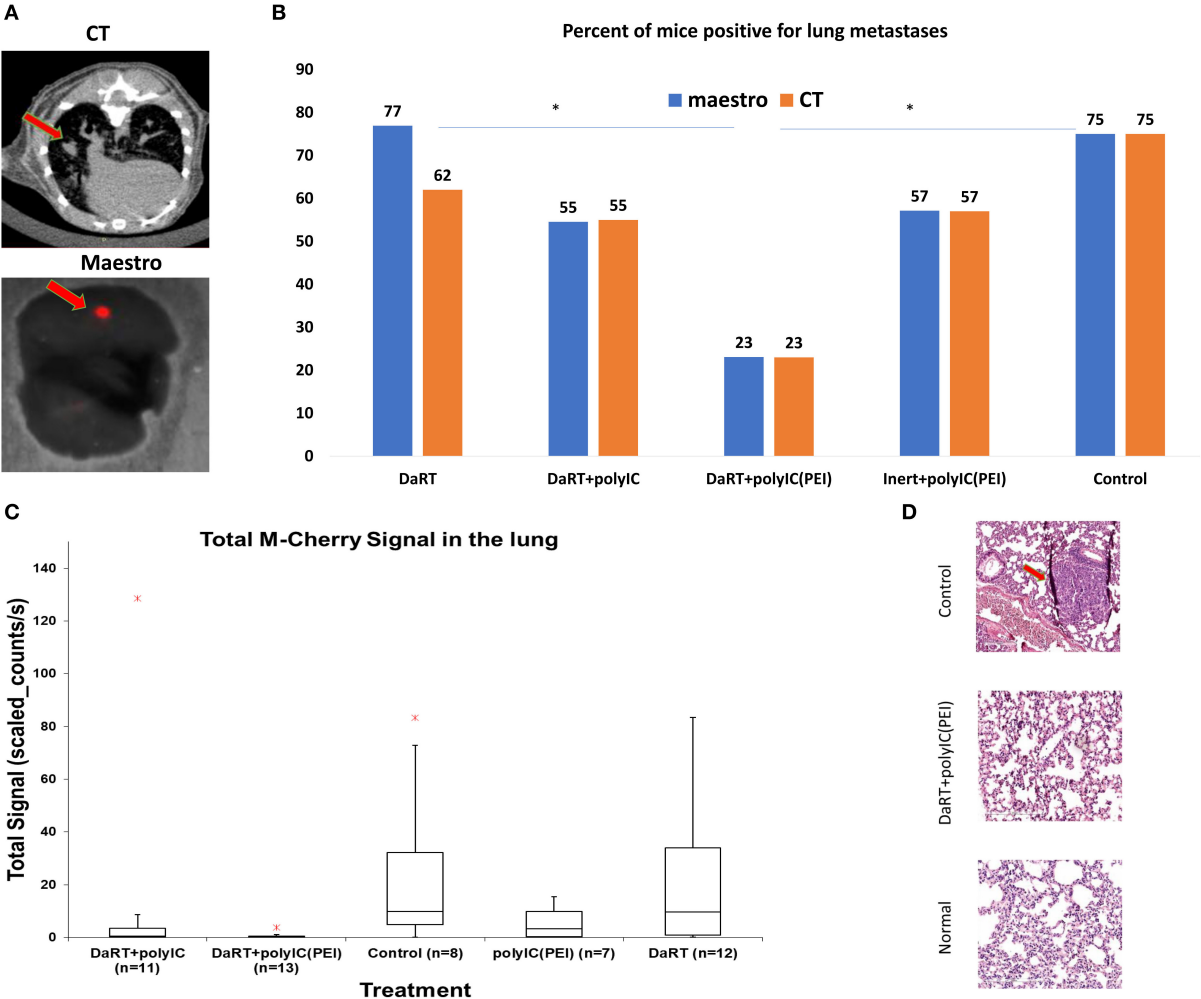
Lung metastases following treatment with DaRT+polyIC^PEI^. 4T1^MCherry^-bearing mice were treated intratumorally with polyIC^PEI^, polyIC^naked^, or vehicle, prior the insertion of a single DaRT seed or an inert seed (see Figure 1A). Lungs were imaged by CT and fluorescence imaging using Maestro, 37 days following tumor cell inoculation. **(A)** Illustrative pictures of metastases as imaged by CT scan or M-Cherry fluorescence imaging. **(B)** Percent of mice that were positive for lung metastases according to CT scan and M-Cherry fluorescence imaging. **(C)** Box plot of M-cherry total signal in the lungs. Red cross denotes an outlier (the graph does not include a DaRT outlier with the value of 362 counts/s). **(D)** Representative histology sections of a normal lung, a lung of a 4T1-bearing mouse treated with DaRT+polyIC^PEI^, and a lung of a 4T1-bearing mouse treated with inert+vehicle. The results are based on cumulative data from two different experiments.

Analysis of lung metastases by CT scan or M-Cherry fluorescence imaging (see Methods, Figure 2A) revealed that 37 days after tumor inoculation, the percent of animals bearing lung metastases was significantly smaller in the polyIC^PEI^+DaRT group (23%) compared to DaRT alone (77%) or inert+vehicle control (75%), *p* (χ^2^ test) <0.05 (Figure 2B). DaRT+polyIC^naked^ also reduced metastatic burden, as demonstrated by total M-Cherry signal in the lungs (Figure 2C). However, a higher number of mice treated with DaRT+polyIC^naked^ were positive for metastases than those treated with DaRT+polyIC^PEI^ (55% compared with 23%). Histology sections of lungs correlated with the findings obtained by M-Cherry and CT (Figure 2D).

### Treatment With Intratumoral polyIC^PEI^ Prior to DaRT Caused Rejection of Panc02 Solid Tumors

The robustness of this treatment was tested by applying it to another aggressive and metastatic tumor model, the pancreatic tumor cell line Panc02. Mice bearing Panc02 tumors were treated with polyIC^PEI^ (25 μg/50 μl), followed by the insertion of a DaRT seed (75 kBq). On the seed insertion day, average tumor volume was ~35 mm^3^.

**Figure 3:**
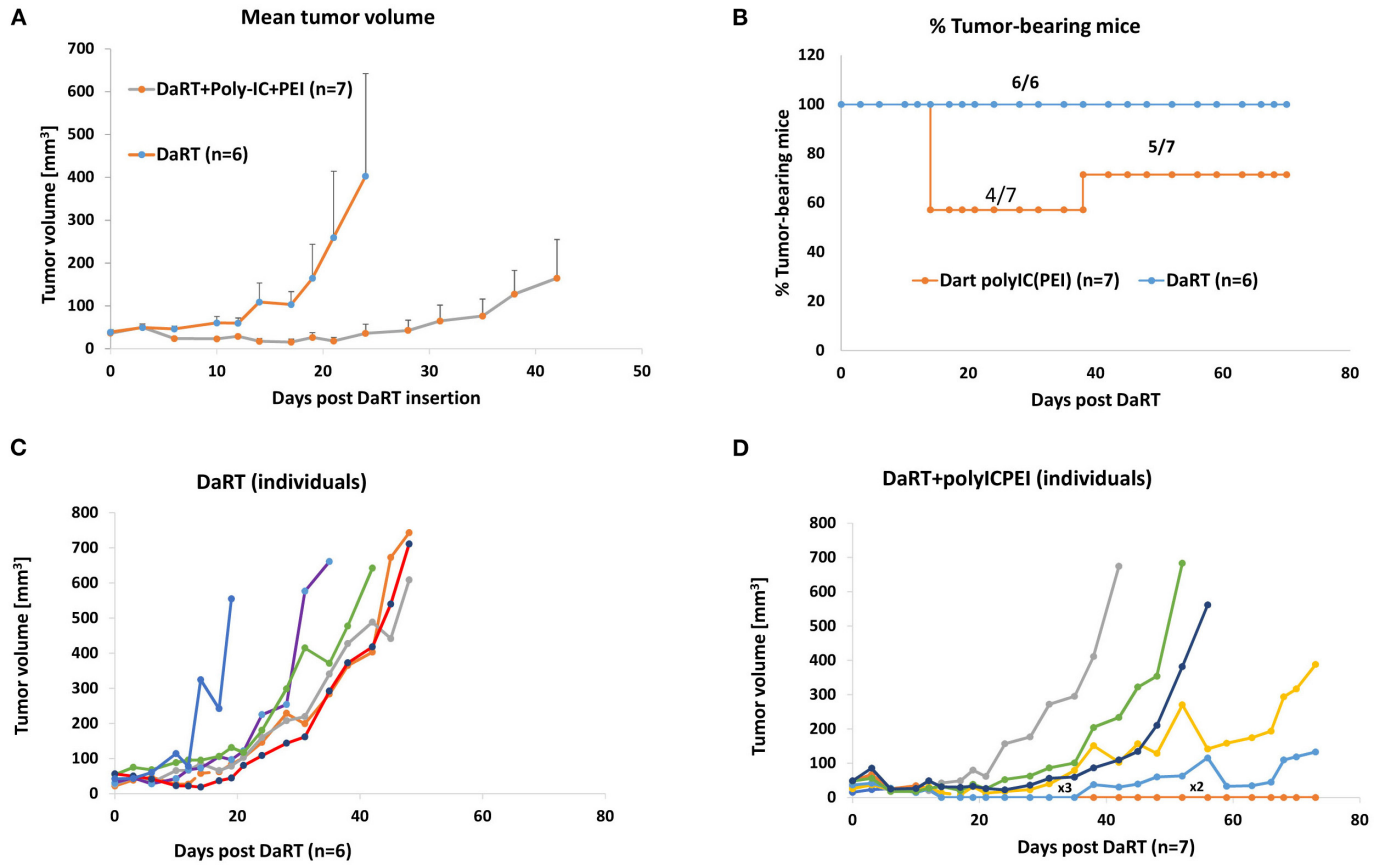
DaRT+polyIC^PEI^ treatment rejected Panc02 tumors. Panc02-bearing mice were treated with an intratumoral injection of polyIC^PEI^ (25 μg /50 μl) 72 and 24 h prior DaRT seed insertion (activity=75 kBq). **(A)** Tumor volume ± SEM. **(B)** The percent of tumor-bearing mice. **(C,D)** Individual tumor development of mice treated with DaRT+polyIC^PEI^ (left) or DaRT+vehicle (right). Each line represents an individual mouse. “x3” denotes a line representing three individuals; “x2” denotes a line representing two individuals.

DaRT+polyIC^PEI^ significantly retarded tumor growth compared with DaRT (12-fold change on day 24 post-DaRT) (Figure 3A). Moreover, the treatment caused tumor rejection in 42.9% (3 out of 7) of the animals for up to 38 days following DaRT upper panel (Figure 3B). At this timepoint, one tumor recurred and 2 out of 7 mice remained tumor-free and survived from this timepoint on, with no signs of illness. Tumors that were not rejected developed more slowly in the DaRT+polyIC^PEI^ group relative to the DaRT group (Figures 3C,D).

### Splenocytes From Mice Pretreated With Intratumoral polyIC^PEI^ and DaRT Inhibit 4T1 Tumor Development When Adoptively Transferred to Naïve Mice

The results above showed that even though polyIC^PEI^+DaRT therapy was administrated locally, at the primary tumor site only, it led to both long-term tumor growth retardation and clearance of distant metastases. The Winn assay was employed to investigate whether the treatment activated a long-term systemic immune memory against tumor antigens. In this *in vivo* cytotoxic test, splenocytes from treated mice or from naïve mice are adoptively transferred to naïve mice in combination with tumor cells, and tumor development is monitored.

**Figure 4:**
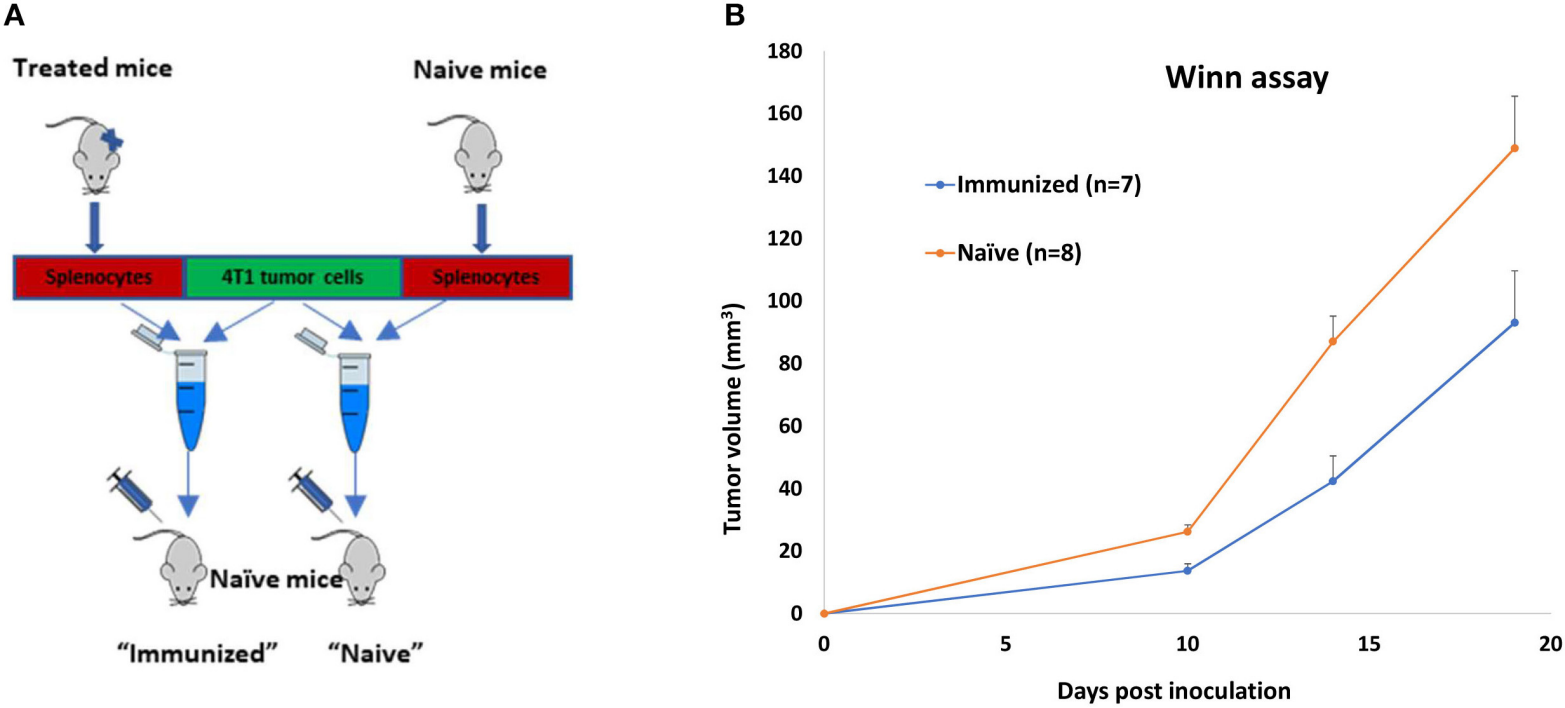
Long-term immune memory in 4T1-bearing mice treated by polyICPEI+DaRT and surgery. 4T1-bearing mice (*n* = 16) were treated by intratumoral 20 μg/40 μl polyIC^PEI^ or vehicle 72 and 24 h prior the insertion of a single DaRT seed (7 mm, 80 kBq). Twenty-four days after tumor cell inoculation, tumors were resected. Mice surviving 9 months after initial tumor cell inoculation (*n* = 4) or naïve mice (*n* = 4) were sacrificed, and their splenocytes were pooled and mixed with 4T1 tumor cells in the ratio of 100:1 (splenocytes: tumor cells). The combined suspension was inoculated into naïve mice. **(A)** Winn assay scheme. **(B)** Mean tumor volume ± SEM, *p*_t−test_ < 0.05 for immunized vs. naïve groups.

Mice (*n* = 16) bearing 4T1 tumors (30 mm^3^) were treated with polyIC^PEI^+DaRT (as depicted in Figure 1A). Residual tumors were resected 24 days following tumor cell inoculation (at a time in which metastases were already present in the lungs), and animals were observed for long-term survival (namely, metastases-related death). Mice surviving for 9 months after tumor inoculation were considered as cured, and their splenocytes were used for an adoptive cell transfer assay (Winn assay). Autopsy of non-surviving mice confirmed lung metastases in all animals (except one animal which had an inflamed lung without visible metastases). Lymphocytes from the spleens of cured mice (*n* = 4) were harvested, pooled, mixed with 2.5 × 10^5^ 4T1 tumor cells in a ratio of 100:1 (splenocytes: tumor cells), and inoculated into naïve mice. Splenocytes of naïve mice (*n* = 4) served as control (Figure 4A).

Splenocytes of treated mice significantly retarded tumor development compared to splenocytes of naïve mice (Figure 4B). A significant reduction in tumor size lasted for 19 days after co-inoculation with tumor cells (52% reduction was evident 14 days after co-inoculation, p_t−test_= 0.002: 42 ± 8 and 87 ± 8 mm^3^ for immune vs. naïve splenocytes, respectively), demonstrating that the treatment induced a long-term antitumor immune memory that is efficient even 9 months following the initial tumor cell inoculation.

### Systemic Low-Dose Cyclophosphamide Combined With Intratumoral polyIC^PEI^ Synergizes With DaRT in Preventing Lung Metastases-Related Death

**Figure 5:**
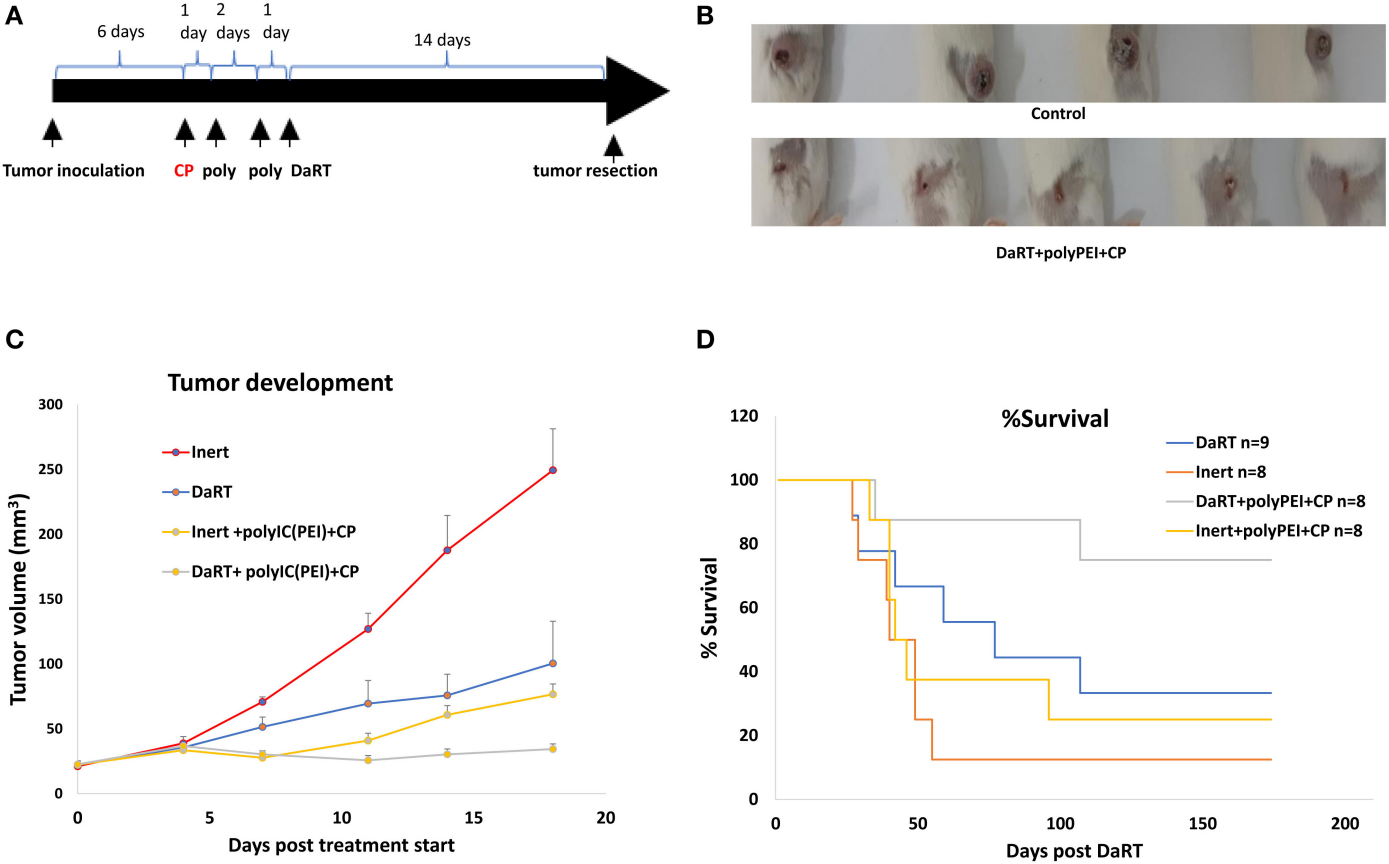
The effect of systemic low-dose CP in combination with local polyIC^PEI^+DaRT on tumor development and metastasis-related death. **(A)** Schematic representation of the treatments with low-dose cyclophosphamide combined with DaRT+polyIC^PEI^ and tumor resection. **(B)** Representative tumors on the day of tumor resection. **(C)** Mice were treated with CP (100 mg/kg, i.p.) combined with polyIC^PEI^ (30 μg/60 μl i.t.) + DaRT (activity = 85 kBq). Presented are tumor volume ± SEM. *P*_t−test_ < 0.05 for DaRT+polyIC^PEI^+CP compared all other treatments. **(D)** Kaplan–Meier survival curves of tumor-resected mice following treatment. *P*_log−ranktest_ < 0.01; < 0.05, for DaRT+ polyIC^PEI^+CP vs. inert+vehicle control or polyIC^PEI^+CP, respectively.

Next, it was investigated whether systemic immunomodulation could further augment tumor growth retardation caused by polyIC^PEI^+DaRT treatment or prolong mouse survival by preventing metastasis-related death. To reduce the number of T regulatory cells (Tregs), a previously proven treatment regimen of low-dose CP was employed (49). 4T1-bearing mice were treated with CP 1 day before the first polyIC^PEI^ injection (30 μg/60 μl), which is 4 days prior to DaRT insertion (activity = 65 kBq), at a time in which tumor size was ~24 mm^3^. DaRT+polyIC^PEI^+vehicle served as a control. Tumor development was followed for 14 days after DaRT insertion, and the tumors were resected thereafter (Figure 5A). In order to examine the effect of the treatments on lung metastases in the above treated animals, monitoring was done for metastases-related death for ~6 months post DaRT, and animal death and the presence of lung metastases were recorded.

Adding CP to DaRT+polyIC^PEI^ treatment significantly reduced tumor volume on the day of tumor resection (47 ± 5, 29 ± 3 mm^3^, *p*_t−test_ < 0.05). The manual measurements of tumor dimensions by a caliper were corroborated with M-cherry fluorescence imaging of the resected tumors. This analysis confirmed that total M-cherry signal and the tumor area (according to M-Cherry fluorescence) where smaller in the polyIC^PEI^+DaRT+CP group compared to the polyIC^PEI^+DaRT group (83 ± 23 vs. 693 ± 280 scaled counts/s, *p*_t−test_ = 0.051; 65 ± 14 vs. 163 ± 20 mm^2^, *p*_t−tes_*t* < 0.005, respectively).

Adding CP to polyIC^PEI^+DaRT treatment extended the survival period relative to treatment with polyIC^PEI^+DaRT without CP. During the 143 days post DaRT insertion, 100% of the animals treated with polyIC^PEI^+DaRT+CP were still alive, at the same timepoint only 71% of the mice treated with polyIC^PEI^+DaRT survived. Nonetheless, the difference between the groups according to log-rank test was not significant and at the end of the experiment identical survival rates (71.4%) were observed in both groups.

Next, an additional experiment was conducted to explore the contribution of DaRT alone (85 kBq) or immunotherapy alone by CP+polyIC^PEI^ (30 μg/60 μl i.t.) relative to the combined treatment. PolyIC^PEI^+CP+inert seed or DaRT+vehicle significantly retarded tumor development compared to inert+vehicle control (*p*_t−test_ < 0.05, on the day of resection) (Figures 5B,C). The combined treatment using DaRT+polyIC^PEI^+CP was significantly more effective than all other treatments (Figure 5C). Ten days post DaRT, for example, tumor volume in the control (inert+vehicle) group was 2.5-fold higher than DaRT only group (DaRT+vehicle), and 3.1-fold higher than that in the immunotherapy-only group (polyIC^PEI^+CP+inert). On that same day, the control group was 6.2-fold higher than the combination treatment (DaRT+polyIC^PEI^+CP), which is more than the expected additive effect (5.6-fold).

At the end of the follow-up period, 75% (6/8) of the mice treated with DaRT+polyIC^PEI^+CP survived, whereas lower survival rates were obtained by DaRT+vehicle (33%, 3/9), inert+polyIC^PEI^+CP (25%, 2/8), or inert+vehicle (12%, 1/8) treatments (Figure 5D). Autopsies of non-surviving animals confirmed metastasis-related death. The effect of DaRT+polyIC^PEI^+CP was significant compared to inert+vehicle (*p*_log−rank test_ < 0.01) and compared to inert+polyIC^PEI^+CP (*p*_log−rank test_ < 0.05), but not compared to DaRT alone (*p*_log−rank test_ =0.081).

### Systemic Low-Dose Decitabine Combined With DaRT Retarded the Growth of 4T1 Tumors

It was then investigated whether systemic low-dose administration of the epigenetic drug decitabine (37), which is known to activate RLR, will strengthen tumor growth retardation induced by DaRT, similar to locally administered polyIC^PEI^. This question is of special therapeutic importance, because decitabine can be administrated systematically to patients.

**Figure 6:**
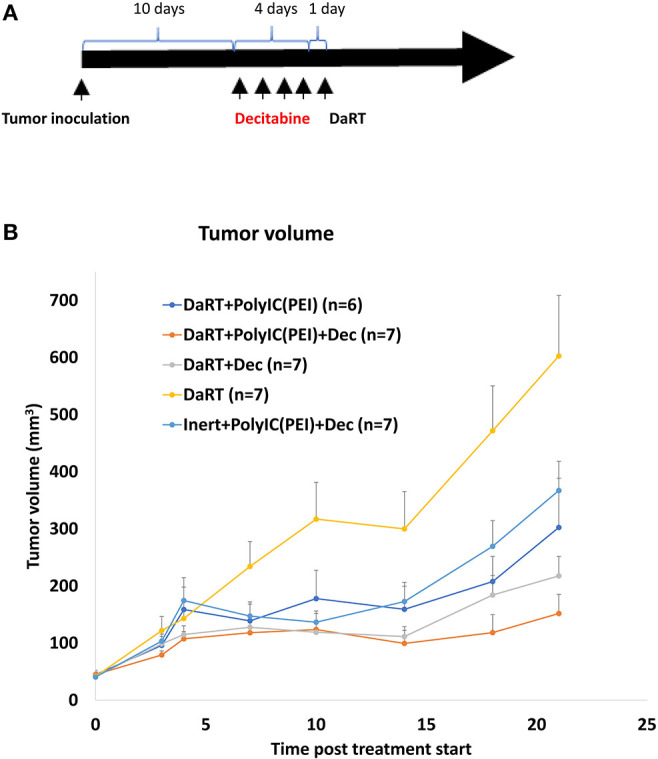
Effect of DaRT combined with decitabine and/or polyIC^PEI^ on 4T1 tumor development. Mice bearing 4T1 tumors (40 mm^3^) were treated with a DaRT seed (75 kBq) in combination with decitabine (1 mg/kg i.p. for 4 consecutive days prior to DaRT) and/or polyIC^PEI^ (30 μg/60 μl, 72 h prior to DaRT and 50 μg/100 μl polyIC^PEI^, 24 h prior DaRT). **(A)** Schematic representation of the treatment by decitabine. **(B)** Presented are the tumor volumes ± SEM.

Mice bearing 4T1 tumors (40 mm^3^) were treated with polyIC^PEI^ (30 μg/60 μl 72 h prior to DaRT and 50 μg/100 μl 24 h prior to DaRT) and/or decitabine (1 mg/kg i.p. daily for 4 consecutive days) prior to the insertion of a DaRT seed (activity = 75 kBq) (Figure 6A).

DaRT combined with decitabine significantly reduced tumor size by 64% compared to DaRT alone, similar to the 50% reduction achieved by DaRT+polyIC^PEI^ (Figure 6B). DaRT combined with both decitabine and polyIC^PEI^ achieved the strongest effect (75% reduction compared with DaRT alone, *p*_t−test_ = 0.001), yet it was only marginally better than DaRT with each stimulator alone. In addition, it was demonstrated that DaRT+decitabine+polyIC^PEI^ was significantly stronger (2.5-fold) compared to the same treatment with a non-radioactive seed (Figure 6B). These results were confirmed in an additional experiment in which mice were bearing larger tumors (85 mm^3^ at the day of treatment start). Tumor volume determined at the same timepoint for inert or DaRT, combined with polyIC^PEI^ and decitabine, was 194 ± 25 vs. 115 ± 16 mm^3^, respectively, *p*_*t*−*test*_ < 0.05).

### DaRT Combined With Systemic Low-Dose Decitabine or Intratumoral polyIC^PEI^ Inhibited the Growth of SQ2 Solid Tumors and Induced Antitumor Immune Response Against Tumor Cell Re-challenge

To further test the robustness of these treatment regimens, including their ability to induce an antitumor systemic immune memory, a tumor model of squamous cell carcinoma (SCC), SQ2, was investigated. SCC was the first type of tumor for which DaRT was tested in human patients (50). SQ2-bearing mice were treated with DaRT (85 kBq) combined with polyIC^PEI^ (25 μg /50 μl), decitabine, or both. Residual tumors were resected 24 days after DaRT, and mice were subjected to tumor re-challenge of the same number of cells (5 × 10^5^ tumor cells), 22 days after tumor resection.

**Figure 7:**
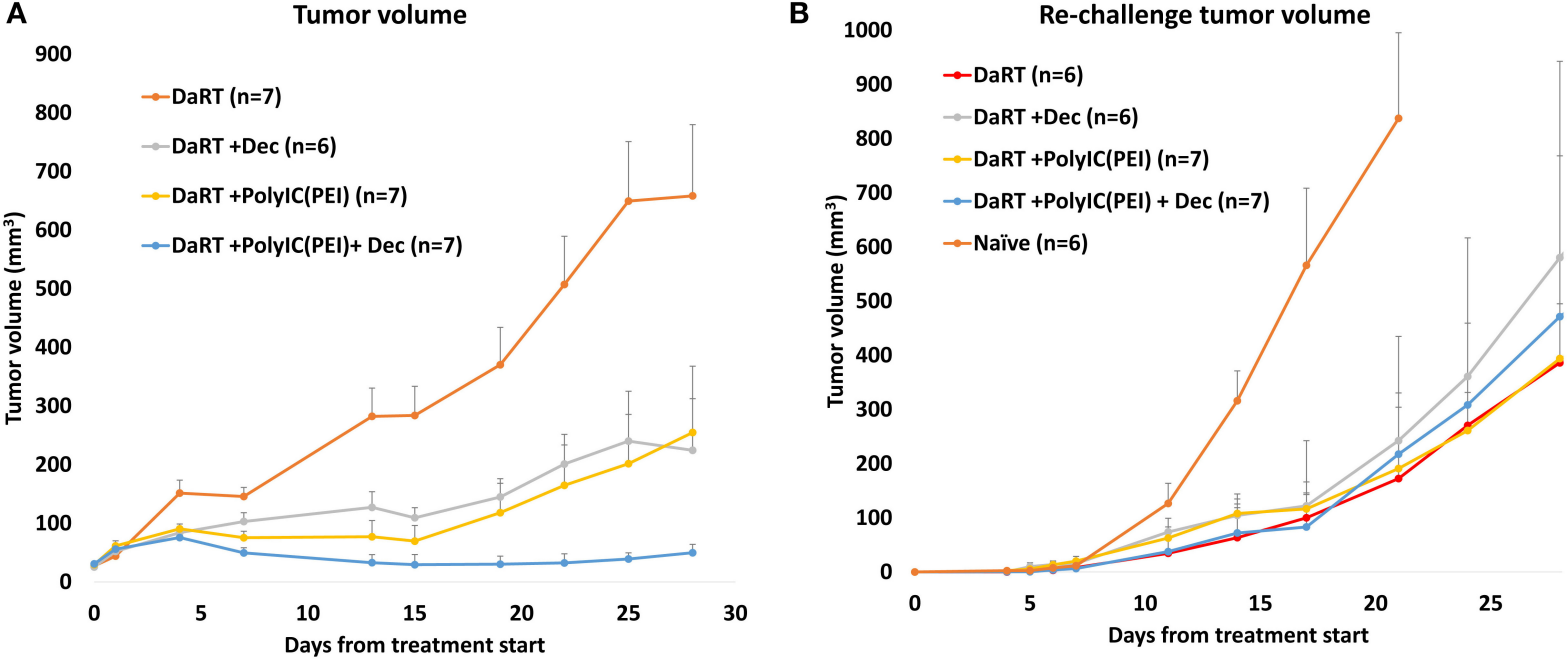
Tumor control and long-term immune memory following treatment with DaRT, decitabine, polyIC^PEI^, or both, in SQ2 tumor model. **(A)** SQ2-bearing mice (30 mm^3^ on first decitabine dose day) were treated with decitabine (1 mg/kg i.p. daily for 4 consecutive days prior to the DaRT) and/or an intratumoral injection of polyIC^PEI^ (25 μg/50 μl 72 and 24 h prior to DaRT). A DaRT (activity=85 kBq) seed was inserted into the tumor 24 h later. Presented are the tumor volumes ± SEM. *P*_t−test_ < 0.05 for all treatments vs. DaRT and for DaRT+decitabine+polyIC^PEI^ vs. DaRT+decitabine. **(B)** Residual tumors were resected 24 days after DaRT and mice were subjected to tumor re-challenge of 5 × 10^5^ tumor cells 21 days after tumor resection. Presented are the tumor volumes ± SEM of the re-challenged tumors. Significant difference was observed for all groups vs. naïve mice (*P*_t−test_ < 0.05, on day 17). *On day 17 one mouse from the DaRT+decitabine+polyIC^PEI^ group died from unknown reason and was not included in the mean tumor volume calculation from this time point on.

DaRT combined with polyIC^PEI^, decitabine, or both significantly retarded tumor development compared to DaRT alone. DaRT+decitabine significantly retarded tumor development similar to DaRT+polyIC^PEI^, leading to a ~65% reduction in tumor size compared to DaRT+vehicle treatment, for up to 27 days from treatment initiation. In this tumor model, the combination of DaRT+polyIC^PEI^+decitabine provided the best results with 92% reduction (20-fold change) compared to DaRT alone and was significantly superior to both DaRT+decitabine or DaRT+polyIC^PEI^ (Figure 7A). DaRT combined with polyIC^PEI^, decitabine, or both preserved the ability to induce long-term immune memory, as demonstrated by a significant reduction (~80%) in tumor size after re-challenge, compared to naïve mice inoculated with the same number of tumor cells (Figure 7B).

## Discussion

In the present study, we examined the possible synergy between the activation of cytoplasmatic dsRNA sensors and tumor ablation by intratumoral diffusion of alpha emitting atoms, both at the local and systemic levels. Treatment with DaRT in combination with cytoplasmatic delivery of polyIC synergistically retarded the development of mouse TNBC tumors and demonstrated rejection of mouse pancreatic tumors. Although the treatment was administrated locally, it also reduced the metastatic load in the lungs and induced a long-term systemic antitumor immune response. Low-dose CP, which was previously shown to reduce the number of Tregs (49), enhanced the tumor control achieved by the local treatment and led to high long-term survival rates that confirmed the reduction in metastatic load.

DaRT-related antitumor immunity (16) was previously attributed to the *in situ* dispersion of tumor antigens, processed by APCs (4). Addition of TLR agonists (16–18) that activate APCs enhanced DaRT's effect. In the current study, combining DaRT with polyIC, complexed with the delivery reagent PEI (PolyIC^PEI^), led to more robust solid tumor control and greater clearance of metastases relative to the same treatment with polyIC only (a TLR3 agonist by itself). This finding suggests that polyIC^PEI^ may exhibit a dual effect, both augmenting antigen presentation by tumor cells (via RLR) and antigen presentation by dendritic cells (via TLR).

The use of DaRT after polyIC^PEI^ may consequently lead to the release of DAPMs after DNA damage, pathogen-associated molecular patterns (PAMPs) from radiation killed cells containing dsRNA, and a massive amount of tumor antigens in the context of MHC class I. This may support important processes such as cross-presentation and cross-dressing (51). In addition, the potential elevation of MHC class I on tumor cells by PolyIC^PEI^ (34) prior to cell death by DaRT may increase the probability to present yet non-presented tumor antigens in the context of MHC class I. Thus, it can be speculated that PolyIC^PEI^-treated, and alpha-radiation-killed, tumor cells may release such MHC class I-antigen complexes, which can be picked up by DCs that in turn present them to CD8+ T cells and help to expand the number of clones recognizing tumor antigens.

In this study, it was shown that DaRT combined with different types of agents known to activate RLR achieved robust antitumor effects in three tumor models. Low-dose decitabine resulted in tumor retardation, similar to polyIC^PEI^. In the SCC tumor model, adding decitabine, polyIC^PEI^, or both reduces tumor size compared to DaRT, yet in the challenge assay, the addition of RLR activation did not affect the power of the long-term immune response relative to DaRT alone (Figure 7B). This may be due to the relatively high number of tumor cells used in the assay. Another possibility is that cells inoculated in the challenge assay were not subjected to a treatment that elevates antigen presentation before inoculation. Namely, antigens that were potentially unmasked by RLR activation *in situ* were not presented by the tumor cells inoculated in the challenge assay, since they were not exposed to the RLR activator and no elevation of MHC class I was induced. Further study is needed to clarify these mechanisms.

The synergy between DaRT and RLR activation can be attributed to additional non-immune-related potential mechanisms. For example, the cellular response to a viral attack may promote transcription related to programmed cell death (52), and thus when DNA damage is induced by alpha radiation, the cellular stress response is already biased to favor cellular death over DNA repair. Indeed, RLR activation by cytoplasmatic delivery of polyIC was found to sensitize tumor cells to ionizing radiation also *in vitro* (53). In the case of decitabine, sensitization to alpha radiation may also be due to chromatin de-condensation (54).

In its first-in-human clinical trial, DaRT was used to treat SCC patients. All patients responded to DaRT, with almost 80% showing complete responses with minor adverse effects (50). In one case, evidence suggests the possible induction of an abscopal effect (55). The treatment regimens presented here efficiently affected both the tumor and distant metastases and extended long-term survival. Low-dose cyclophosphamide, previously found to reduce the number of Tregs, demonstrated the potential of immunomodulating therapies used in clinical practice (56) to further enhance these effects. Taken together, the results presented here may suggest future directions for improved therapeutic protocols for treating patients with metastatic cancer.

## Data Availability Statement

The datasets generated for this study are available on request to the corresponding author.

## Ethics Statement

The animal study was reviewed and approved by Tel Aviv University ethics committee.

## Author Contributions

VD: conceptualization, formal analysis, investigation, methodology, project administration, resources, supervision, validation, visualization, writing—original draft, and writing—review & editing. ME and MS: investigation, methodology, and resources. EG: formal analysis, investigation, visualization, and writing—original draft. FM, AS, AC, and EF: investigation. YZ: investigation and resources. RG: conceptualization and writing—review & editing. IK: conceptualization, funding acquisition, methodology, supervision, and writing—review & editing. YK: conceptualization, funding acquisition, methodology, project administration, supervision, validation, visualization, writing—original draft, and writing—review & editing. All authors contributed to the article and approved the submitted version.

## Conflict of Interest

YK and IK serve as consultants for Alpha Tau Medical LTD. Tel Aviv, Israel. VD, ME, MS, EF, and AS are employees of Alpha Tau Medical LTD. Tel Aviv, Israel. YK, IK, VD, ME, and MS hold stock options in Alpha Tau Medical LTD. Tel Aviv, Israel. YK, IK, and VD are the inventors of a patent application submitted by Alpha Tau Medical LTD, which is related to the results of the current study. The remaining authors declare that the research was conducted in the absence of any commercial or financial relationships that could be construed as a potential conflict of interest.
